# Neglected Bilateral Posterior Shoulder Fracture Dislocation in an Uncontrolled Seizure patient

**DOI:** 10.12669/pjms.314.8231

**Published:** 2015

**Authors:** Moaath A. Amir, Bashir Alenazi, Richard K.H. Wyse, Waleed Tamimi, Omar Kujan, Tajdar Khan, Faris Q. Alenzi

**Affiliations:** 1 Moaath A. Amir, Department of Orthopedics, Military Hospital, Riyadh, Saudi Arabia; 2 Bashir Alenazi, Department of Orthopedics, Military Hospital, Riyadh, Saudi Arabia; 3 Richard K.H.Wyse, Dept. of Surgery, Imperial College London, UK; 4 Waleed Tamimi, Biochemistry Section, Pathology Department, KAMC, Riyadh, Saudi Arabia; 5 Omar Kujan, Alfarabi Dental College, Riyadh, Saudi Arabia; 6 Tajdar Khan, College of Pharmacy, Salman bin Abdulaziz University, Al-Kharj, Saudi Arabia; 7 Faris Q. Alenzi, College of Appl Med Sci, Prince Sattam bin Abdulaziz University, Al-Kharj, Saudi Arabia

**Keywords:** Posterior dislocation, Shoulder joint, Trauma, Seizures

## Abstract

Posterior dislocation of the shoulder is a rare injury that occurs secondary to trauma and seizures. Diagnosis is often missed and treatment is challenging. Neglected posterior dislocation is associated with Hill-Sachs lesion which leads to locking of dislocation. Correct diagnosis is achieved by history taking, a physical examination and appropriate imaging. In neglected shoulder dislocation with uncontrolled seizure and humeral head defects of up to 45% the McLaughlin procedure shows excellent results at follow-up.

## INTRODUCTION

Posterior dislocation of the shoulder joint is a rare injury which occurs in less than 2% of all shoulder dislocations. Misdiagnosis of the injury can occur in more than 50% of patients, most commonly due to an inappropriate physical examination such as prominence of the coracoid, posterior prominence of the humeral and decreased external rotation of the shoulder,^1^,^2^ and/or an inadequate radiographic examination. Appropriate imaging such an axillary or Velpeau view and anteroposterior (AP) views are also important in order to reach the correct diagnosis.^3^ Seizures have been reported in 34% of cases.^4^ The causes of posterior dislocation of the shoulder are usually due to trauma, epilepsy or electric shock. Different management options are described depending on the size of the defect, and the time from dislocation. Treatment options include the McLaughlin procedure, rotational osteotomy or arthroplasty.^5^,^6^ This case report presents a McLaughlin technique using absorbable suture anchors for patients with neglected, locked posterior dislocation of the shoulder.

## CASE REPORT

A 37 years old male soldier known to have uncontrolled status epilepticus due to non-compliance of treatment presented to the emergency room three weeks after having a seizure complicated by undiagnosed right shoulder pain and decreased shoulder external rotation. AP and axillary X-ray views (Fig.1a) were carried out which showed right posterior locked fracture dislocation of the humeral head, whilst a CT scan (Fig.1b) showed a 35% humeral head defect reverse Hill-Sachs lesion (also called a McLaughlin lesion). One week later the patient underwent a right shoulder McLaughlin procedure and shoulder Spica followed by passive, then active-assisted, and active range of motion and rotator cuff strengthening exercises.

**Fig. 1a:**
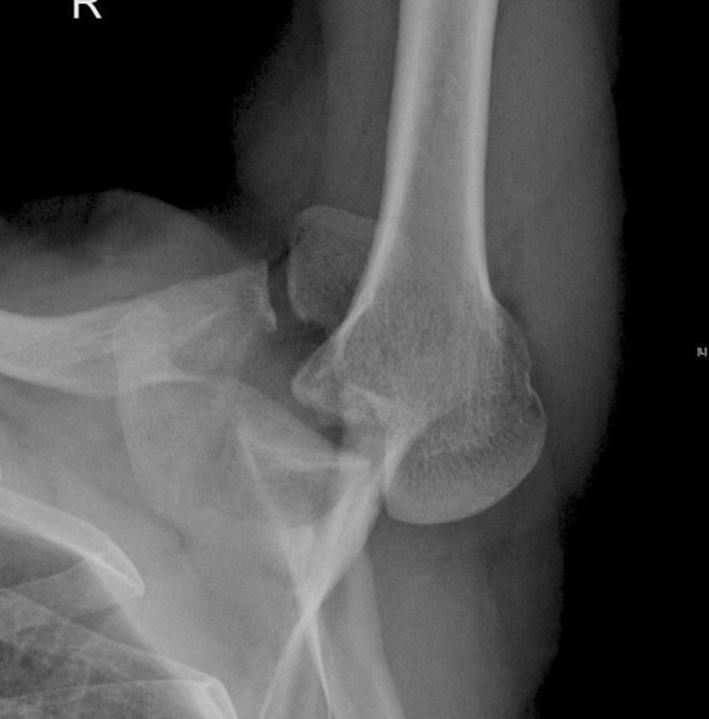
Axillary view shows locked right posterior shoulder dislocation

**Fig. 1b:**
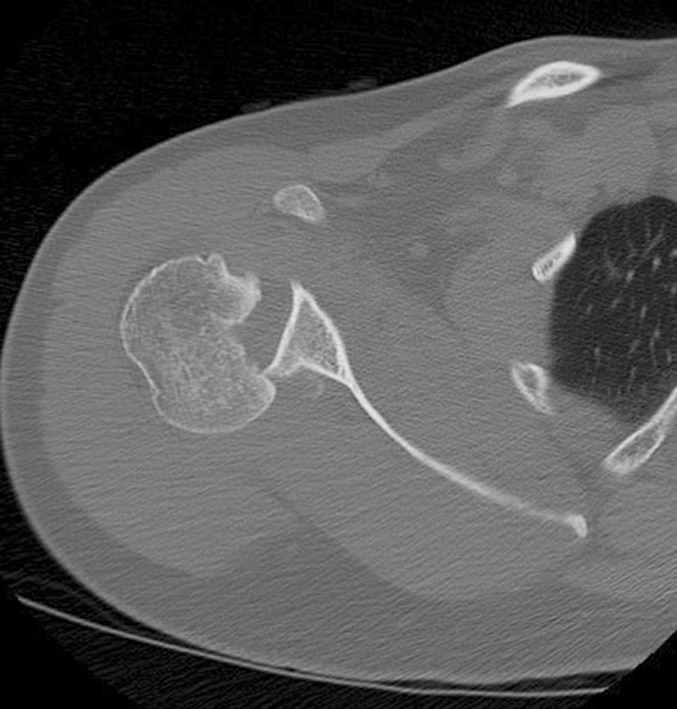
Axial C. T. shows humeral head defect.

Six months later the patient had another seizure which resulted, this time, in a left posterior shoulder fracture dislocation, and was diagnosed and treated in another hospital with close reduction. Due to patient non-compliance to their follow ups and their treatment for epilepsy, the patient had multiple seizures. One month later the patient presented in our ER with left shoulder pain and decreased left shoulder external rotation. X-rays of AP and axillary view (Fig.2a) were conducted which showed left posterior locked fracture dislocation of the humeral head and a CT scan (Fig.2b) showed a 40% humeral head defect reverse Hill-Sachs lesion. The patient underwent a left shoulder McLaughlin procedure and shoulder Spica in external rotation followed by passive, then active-assisted, and active range of motion and rotator cuff strengthening exercises.

**Fig. 2a:**
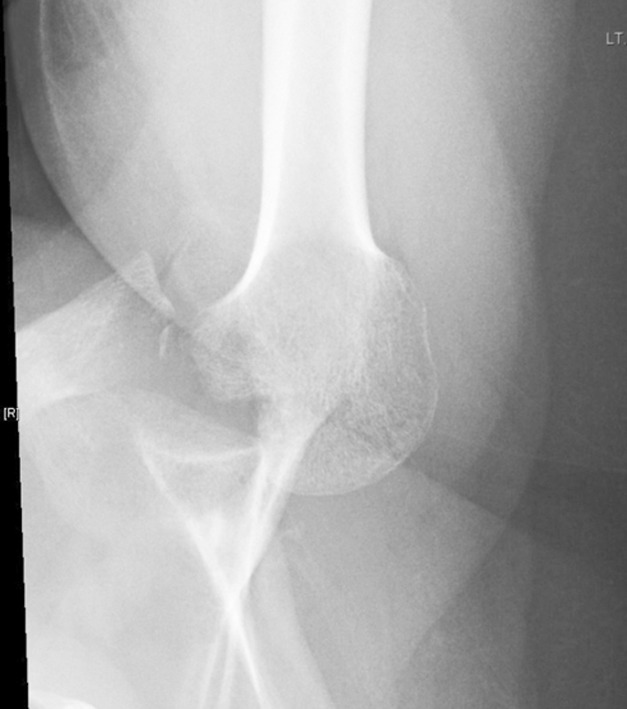
Axillary view shows locked Left posterior shoulder dislocation.

**Fig. 2b:**
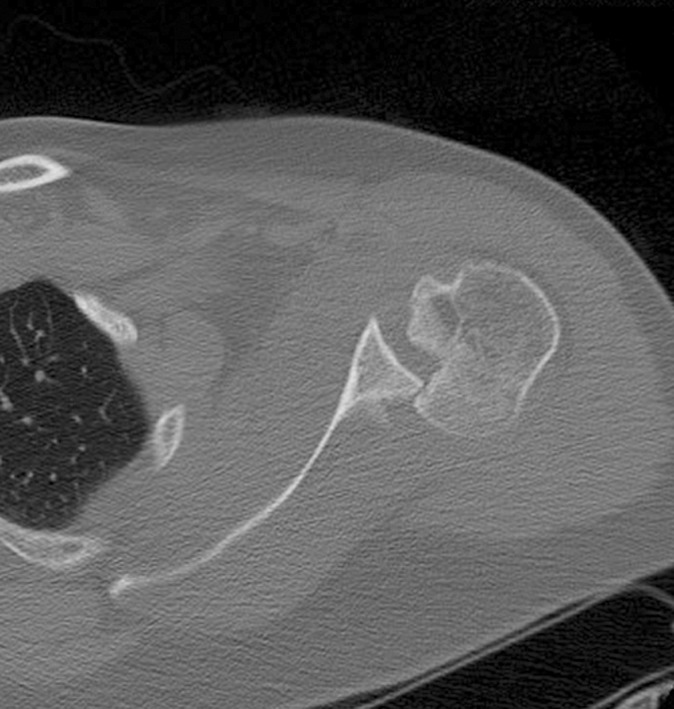
Axial C. T. shows humeral head defect.

## RESULTS

**Fig. 3:**
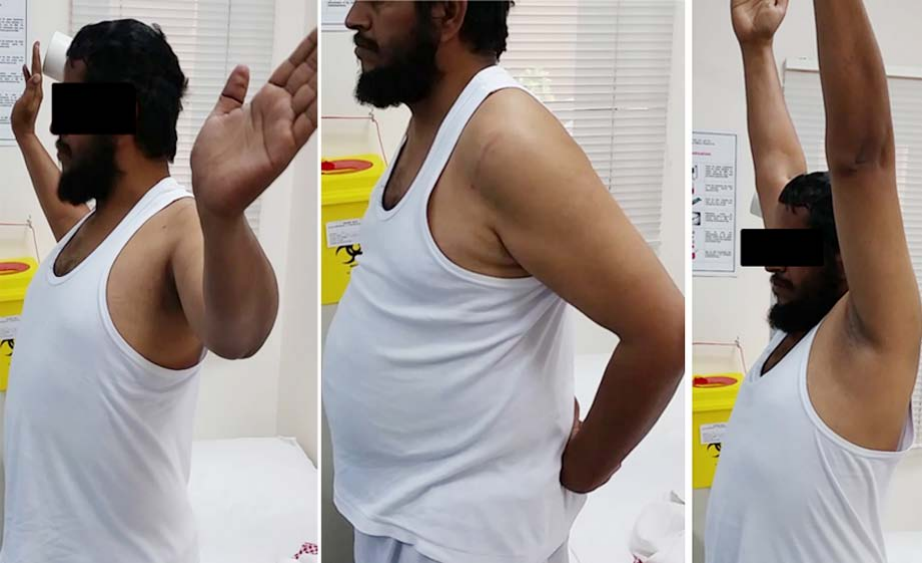
Range of motion of shoulder joint post McLaughlin procedure.

Twenty three months from the right shoulder surgery, and 14 months from left shoulder surgery, and with multiple uncontrolled seizures, the patient has intact, stable and good joint congruency of his bilateral shoulder joints. There is now a pain-free range of motion, external rotation of 45 degrees, intact lifts of test abduction of 170 degrees, and flexion of 160 degrees (Fig.3). The patient returned to his normal lifestyle as well to his hobbies (which are hunting and car mechanics) and has an absolute Constant Score of 86 points for both shoulders.

### Surgical Technique

The patient underwent general anesthesia in a beach-chair position, then a deltopectoral approach was made and the joint was reached. Identification of subscapularis was then made and incised at its tendon. This was followed by thorough irrigation of the joint. The defect was drilled and the incised tendon was sutured in the defect by non-absorbable suture in order to fill the defect. Stability of the shoulder joint was evaluated intraoperatively by examination in all directions of the range of motion with direct observation. The wound was closed in layers.

## DISCUSSION

Posterior shoulder dislocation is a challenging and the key to its accurate diagnosis is by taking appropriate history and by performing an appropriate physical examination on the patient. The importance of taking appropriate radiographs should also be emphasized, especially the axillary or the Velpeau view and, if not possible, to undertake a CT Scan. Attention to adopting an appropriate management technique depends on the defect size in order to try to achieve as much of the pre injury shoulder function as possible.

## CONCLUSION

The presented case underlines a rare situation. Very few patients have been reported with an uncontrolled seizure with poor compliance which has then resulted in a neglected bilateral locked posterior shoulder dislocation which has then been diagnosed after appropriate and careful investigation. In this case, a specific planned McLaughlin technique was performed which resulted in a satisfactory painless stable shoulder and a successful return to a normal lifestyle for the patient.
